# Impact of dataset diversity on accuracy and sensitivity of parallel factor analysis model of dissolved organic matter fluorescence excitation-emission matrix

**DOI:** 10.1038/srep10207

**Published:** 2015-05-11

**Authors:** Huarong Yu, Heng Liang, Fangshu Qu, Zheng-shuang Han, Senlin Shao, Haiqing Chang, Guibai Li

**Affiliations:** 1 State Key Laboratory of Urban Water Resource and Environment (SKLUWRE), Harbin Institute of Technology, 73 Huanghe Road, Nangang District, Harbin, 150090, P.R. China

## Abstract

Parallel factor (PARAFAC) analysis enables a quantitative analysis of excitation-emission matrix (EEM). The impact of a spectral variability stemmed from a diverse dataset on the representativeness of the PARAFAC model needs to be examined. In this study, samples from a river, effluent of a wastewater treatment plant, and algae secretion were collected and subjected to PARAFAC analysis. PARAFAC models of global dataset and individual datasets were compared. It was found that the peak shift derived from source diversity undermined the accuracy of the global model. The results imply that building a universal PARAFAC model that can be widely available for fitting new EEMs would be quite difficult, but fitting EEMs to existing PARAFAC model that belong to a similar environment would be more realistic. The accuracy of online monitoring strategy that monitors the fluorescence intensities at the peaks of PARAFAC components was examined by correlating the EEM data with the maximum fluorescence (*F*_*max*_) modeled by PARAFAC. For the individual datasets, remarkable correlations were obtained around the peak positions. However, an analysis of cocktail datasets implies that the involvement of foreign components that are spectrally similar to local components would undermine the online monitoring strategy.

Dissolved organic matter (DOM) has always been a major concern in natural and engineered systems^1^. Conventional characterization techniques, which generally focus on the bulk characters of DOM, e.g. total organic carbon (TOC), ultraviolet (UV) absorbance, and specific UV absorbance at 254 nm (SUVA), cannot provide further information on DOM fractions^2^. In addition to these traditional techniques, liquid chromatography with organic carbon detector (LC-OCD) and fluorescence spectroscopy are increasingly adopted to characterize DOM^3^,^4^,^5^. Three-dimensional fluorescence excitation-emission matrix (EEM) spectroscopy enables a rapid and sensitive characterization of DOM. The EEM can be correlated to the fluorescence components in the DOM and thus give more insight into DOM fractions and their chemical characteristics^6^,^7^,^8^. Moreover, Parallel Factor (PARAFAC) analysis was proposed to mathematically separate spectrally overlapping EEM data into chemically independent fluorescence components^9^. Studies have adopted PARAFAC analysis of EEMs to characterize DOM in various natural and engineered environments, e.g. marine, fresh water, ground water environments, as well as wastewater, recycled and drinking water systems^10^,^11^,^12^,^13^,^14^,^15^. Some researchers even proposed to develop a universal model that involved samples from aquatic environments as diverse as possible, so that it can be directly fitted to new EEMs obtained from different sources^13^,^16^,^17^,^18^,^19^. Moreover, online monitoring of DOM using EEM-PARAFAC has drawn a lot of attention, and many studies have referred to the possibility of online monitoring with this new technique^13^,^16^,^20^,^21^.

However, EEM PARAFAC has its limitations. It is known that DOM in different aquatic environments incorporates different fluorescence components, and even similar components from different systems can exhibit shifts in locations of fluorescence peaks^13^,^16^. The shifts of componential peaks, either due to a new component introduced by peculiar samples or resulted from a long sampling duration, may lead to systematically biased estimates of the spectrum and score of a component in the PARAFAC model^13^,^16^. Therefore, it was recommended that an EEM dataset for PARAFAC analysis would better contain samples from similar types of sources^13^,^19^. However, because of the samples or components introduced through some unexpected sources (especially when involving a long sampling duration or a contamination event), the discrepancy derived from the variation of sample sources would be inevitable during the PARAFAC modeling^13^,^15^. Therefore, it is necessary to examine the PARAFAC model that incorporates samples from diverse sources and to analyze the actual sensitivity of the bias derived from diversity of the sample set.

In terms of the universal model mentioned above, a large dataset of EEMs that contains a great diversity in DOM source and chemical quality is required to build this universal model. Therefore, if the sample set diversity does impact the accuracy and sensitivity of the PARAFAC model, the universal model will be highly vulnerable. Although PARAFAC models that derived from large datasets (including 307-1479 sample) were successfully developed in some studies^13^,^19^,^22^, the sampling sources in each of these researches focused on only a relatively narrow range of natural or engineered environments. The effect of spectral variability on the accuracy and sensitivity of a PARAFAC model still deserves further investigation.

In terms of online monitoring, a commonly proposed strategy is to monitor key pairs of excitation-emission wavelength at the componential peaks that were determined by PARAFAC modeling^13^,^16^,^21^. This method assumed that the fluorescence overlap is much gentler at the target wavelengths, so that the maximum fluorescence (*F*_max_) of each component in a sample (which is known to be proportional to the concentration of the corresponding component^14^,^22^,^23^) could be estimated quite accurately from the measurement at the excitation/emission wavelengths of the peaks. Murphy *et al*.^13^ as well as Shutova *et al*.^16^ assessed the sensitivity of this strategy, and proved that the fluorescence overlap was very minor at peak points (*R*^*2*^ = 0.90-1.00). But EEMs in their studies were sampled only from recycled water treatment plants and drinking water treatment plants respectively, which undermine the applicability of the result to EEMs from other sources. Moreover, some contaminated samples with fluorescence components derived from other sources are believed to be highly possibly encountered during a long term monitoring, because the monitoring site may access to these multi sources. Therefore, it is necessary to examine the accuracy and sensitivity of the peak monitoring strategy at the multi-source situation or during a contamination event.

The aim of this paper was therefore to investigate the representativeness and sensitivity of a PARAFAC model with a dataset of EEMs stemmed from different aquatic sources and the implications for developing a universal PARAFAC model. Furthermore, whether monitoring fluorescence intensity at the peaks of PARAFAC components was feasible to estimate the *F*_max_ (especially during a contamination event) was also evaluated.

## Results

### Effect of sample set diversity on the accuracy and sensitivity of PARAFAC model

#### Peak location comparison among PARAFAC models

Samples from a river (76 samples), effluent of a wastewater treatment plant (62 samples), and algae excretion (85 samples) were collected and subjected to following analysis. They are named as natural organic matter (NOM), effluent organic matter (EfOM), and extracellular organic matter (EOM) hereinafter. A global dataset that contains all samples was also subjected to PARAFAC analysis and the obtained global model was compared with each individual model.

**Figure 1:**
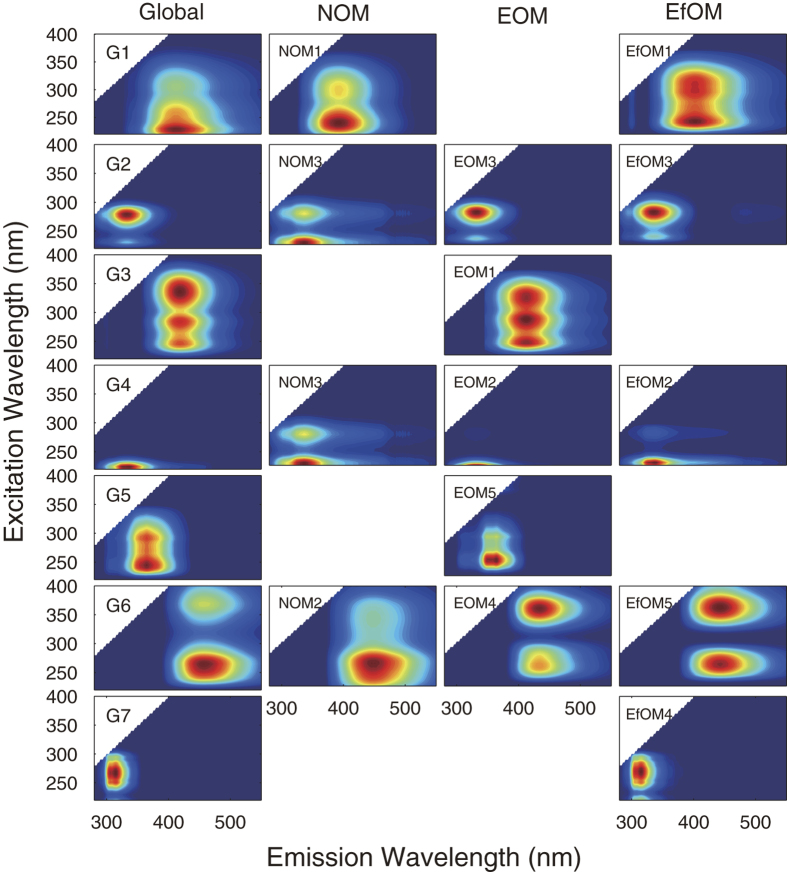
Contour plot of 7 components in Global model and the corresponding components in individual models (components were numbered arbitrarily by the PARAFAC models).

According to the procedures recommended by Murphy *et al*.^24^, 3-7 components were finally identified in the datasets (Fig. 1). All models converged quickly and each was half split validated (Supplementary Fig. S1-6). In the global model, 7 PARAFAC components were identified. The peak locations of these components and the comparison with previously identified components in published studies are listed in Table 1. The sources of the samples in published models are also listed for comparison. Tyrosine-like (G7) and tryptophan-like substances (G2 and G4) are common to practically all published models, and these protein-like substances can be found in almost all different sources. But at times G2 and G4 were merged into one component in published research^25^. Similarly, G1 and G6 are also commonly referred to humic-like components. G5 was relatively rarely reported, and was mainly founded in surface water and algal secretion^14^,^26^. G3 was almost exclusively found in the published models encompassing algal excretion samples^26^,^27^.

As shown in Fig. 1, components in global model encompassed all components in individual models, and no new component was identified in individual models. Components G2, G4, G6 were identified in all models. G2 and G4 were combined in NOM model. G3 and G5 were unique to EOM, while G7 was only found in EfOM. This demonstrated the ability of PARAFAC modeling to identify the peculiar components from some different sources. As shown in Fig. 1, although all components in the global model can be matched with the similar components in the individual models, the peak locations of some similar components were subtly shifted in different models.

**Figure 2:**
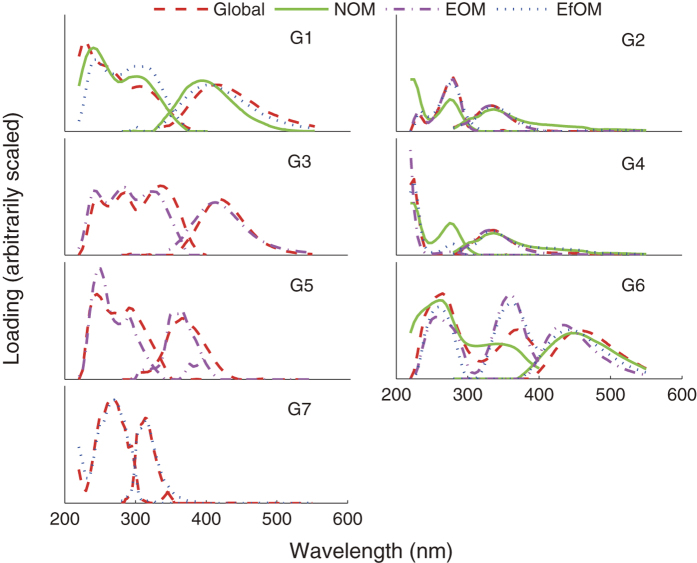
Comparison of excitation and emission loadings of PARAFAC components in different models (excitation to the left of emission spectra).

Figure 2 shows the variation in peak location and/or spectral shape of the components in different models. The shapes of G2 and G4 in NOM model were distinctly different from others. This can be attributed to that the G2 and G4 were combined in NOM model. But it can be seen that the peak locations of G2 and G4 in NOM model were relatively similar to others. Differences in location were also observed for components G1 and G6, with spectra shifts up to ~20 nm observed in both excitation and emission spectra.

The tucker congruence coefficients (*r*_*c*_) of similar components in global model and individual models are listed in Table 2. This quantitative analysis confirmed the observations before. *r*_*c*_ of G2 and G4 in NOM model were quite low (*r*_*c*_ < 0.85). This can be attributed to the inability of NOM model to distinguish component G2 and G4. G1 in NOM and EfOM models, as well as G6 in all three individual have relatively low *r*_*c*_ (0.85 < *r*_*c*_ < 0.95). The rest G3, G5 and G7 components have *r*_*c*_ larger than 0.95, which represents a striking similarity between the corresponding components in global and individual models.

It can be easily found that component G1, G2, G4 and G6 have more than one similar component in individual models, while components in global model that have only one similar component in individual models always have a high *r*_*c*_ (i.e. G3, G5 and G7). It can be concluded that PARAFC modeling is able to accurately identify distinguishing components with almost no peak shift; however, when modeling a dataset contained similar components with a subtle peak shift, the global PARAFAC model will treat them as a same one, but the peak shift can distort the component identified in the global model.

#### *F*_max_ correlation among PARAFAC models

The peak locations and spectrums of components determined in PARAFAC model directly affect the decomposition of an EEM in the modeling. According to the definition of PARAFAC analysis, if a peak shift (or a spectrum variation) happens to a component, the final *F*_*max*_ calculated can be considerably varied. Moreover, because of the interdependence of the simultaneously estimated components in a PARAFAC model, the inclusion of one or more poorly estimated components can even significantly affect the spectra and *F*_*max*_ of other components.

Liner regressions of component *F*_*max*_ of same samples in the global against those in individual models were conducted. The correlation coefficient (*R*^*2*^) and regression coefficient (*m*) of corresponding individual and global components are listed in Table 3. *R*^*2*^ values of G1 in EfOM and G2 in NOM and EOM were low (<0.85), while *R*^*2*^ values of G1 and G2 in other individual model were relatively high (>0.9). It can be also found that G3, G5 and G7, which did not have similar components from other sources with their excitation (Ex) and emission (Em) loadings accurately estimated in the global model, have extremely high *R*^*2*^ values (>0.95). Therefore, the similar components from different sources involved in the global model impacted the sensitivity and accuracy of that model. They were regarded as the same component in the global model, and the Ex/Em loadings as well as *F*_*max*_ of these components obtained were biased. However, G4 and G6 in global model also exhibited a poor estimation of Ex/Em loadings, but the *F*_*max*_ of these components obtained from global model were relatively accurate (*R*^*2*^ > 0.9). This means a poor estimation of Ex/Em loading in a PARAFAC model is not necessary to result in a poorly estimated *F*_*max*_. However, the poor correlation of *F*_*max*_ between global and individual models for some components means that the alternative PARAFAC models (global and individual models) are not interchangeable in estimating the intensities of some problem components.

The regression coefficients (*m*) were also calculated. As shown in Table 3, the *m* values are far from 1.0 in some regressions, although the corresponding *R*^*2*^ values are relatively high. It resulted from the different Ex Em loadings of a component resolved in different models. However, this departure should not be bothered. Because in DOM samples the *F*_*max*_ cannot be convert to concentrations, it can be only used for relative quantification. As long as *F*_*max*_s of a component in a sample modeled in different PARAFAC models are highly correlated (with high *R*^*2*^ value), both of these *F*_*max*_ values can be used for the relative quantification.

#### Correlation between fluorescence intensity at the peak and Fmax

To assess the accuracy and sensitivity of the peak monitoring strategy, *F*_*max*_ of each component was regressed against the fluorescence intensities in the original EEMs. This analysis was done for each individual dataset (i.e. NOM, EOM, EfOM). In order to model a contaminant event, e.g. river sample contaminated by algal excreta or effluent of wastewater treatment plant, two mixture datasets (76 NOM samples + 10 EOM samples and 76 NOM samples + 10 EfOM samples) were constructed, and subjected to PARAFAC analysis and the correlation analysis described above.

**Figure 3:**
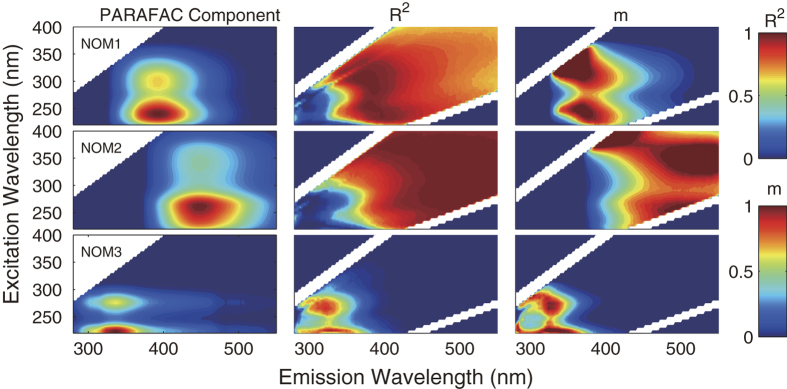
Contour plot of each component, and correlation coefficient (*R*^*2*^) and regression coefficient (*m*) obtained via linear regression (*F*_*max*_ against original fluorescence intensity) for each component in the NOM model.

According to the linear regression analysis, the *R*^*2*^ was close to 1.0 around the peak location for components in all individual models (Fig. 3 and Supplementary Fig. S7-8). The *R*^*2*^ values at the peak locations in different individual models are shown in Table 4 and Supplementary Table S1-2. All the *R*^*2*^ values are higher than 0.9, which indicated a striking correlation. This means the overlapping of fluorescence intensity from different components was less occurred around the peak area. Therefore, the *F*_*max*_ of a component in a sample can be accurately estimated by the fluorescence intensity measured at the peak. Moreover, even if a subtle peak shift is encountered, the fairly big high *R*^*2*^ area shown in Fig. 3 and Fig. S7-8 indicates a still accurate estimation of *F*_*max*_.

The regression results of the cocktail dataset shown in Fig. S9 and S10 and Table 4 indicated a relatively weak correlation between *F*_*max*_ and fluorescence intensity. As shown in Table 4, at the peaks of NOM3 in NOM + EOM model and NOM 1, 2, and 3 in NOM + EfOM model, *F*_*max*_ did not strongly correlated with the peak intensity (*R*^*2*^ < 0.85). As shown in Fig. 1,2, components in EOM and EfOM models that do not exist in NOM model are obviously overlapped with NOM components, e.g. EOM5 & NOM3, EOM4 & NOM3. Therefore, the relatively lower *R*^*2*^ found in the mixed model can be attributed to the spectral overlap between the intrusive components and the original components.

Similar to the explanation for the *m* value in Table 3, the value of *m* of *F*_*max*_ against fluorescence intensity detected in Table 4 needs not to be minded. As long as *R*^*2*^ value in current regression is high, the measured fluorescence intensity at the peak can be correlated to the *F*_*max*_, and in turn, correlated to the concentration of the corresponding component.

## Discussion

**Figure 4:**
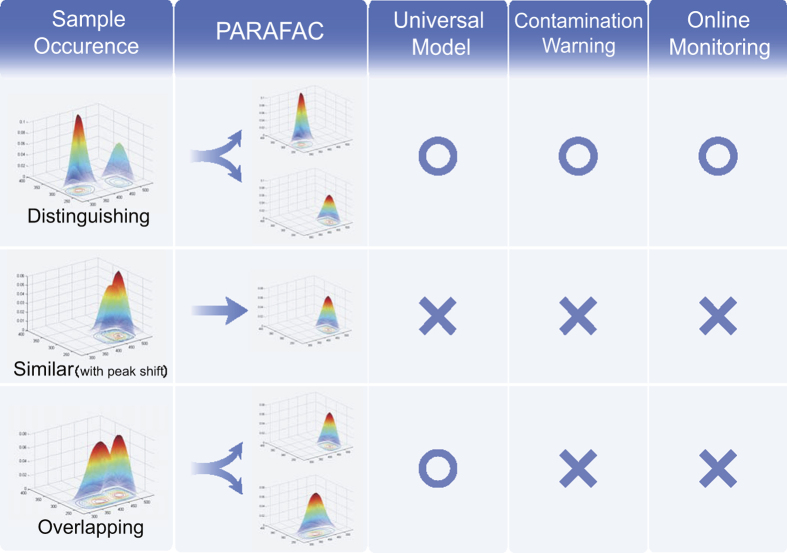
Schematic diagram of the feasibility of developing universal model, contamination warning, and online peak monitoring under different occurrences of componential spectroscopy

According to the results above, it can be concluded that when involving diverse sample sources, the PARAFAC model can successfully decompose different components. The distinguishing components from peculiar sources (e.g., G3, G5, and G7) can be discriminated, while the similar components from different sources will be treated as single component (e.g. G1, G2, G4, and G6). However, the peak shift occurred between similar components from different sources (G1 and G2) undermined the sensitivity and representativeness of the model. Therefore, a spectral variation of a component derived from the diversity of sampling source is believed to impact the development of a global PARAFAC model. All the occurrences discussed above are summarized in Fig. 4. Although the dataset obtained in current research is far from big and diverse enough to construct a universal PARAFAC model as discussed by Fellman *et al*.^19^, the bias found in the global PARAFAC model in current research could be extrapolated to that proposed universal model. Some published studies also showed this point^13^,^16^. It was found that the spectral variation derived from differences of treatment processes and raw water contributed to PARAFAC’s difficulty. Fellman *et al*.^19^ also found that samples from different sources caused a problem when fitting a new sample set to an existing PARAFAC model. Therefore, it is considered that building a universal PARAFAC model that could be widely available for directly fitting new EEMs would be quite difficult, but fitting new EEMs to existing validated PARAFAC model that all belong to a narrow aquatic environment would be more realistic^19^,^23^.

The ability of PARAFAC modeling to discriminate the distinguishing components from peculiar sources implies that EEM-PARAFAC analysis is able to identify a contamination event and serve as an early warning strategy, but the new component introduced (or contamination indicator) must be spectrally different from the existing components (without spectral overlap) (as shown in Fig. 4). Because of the rapidness and high sensitivity of this method, fluorescence monitoring has been proposed to be applied in the cross-connection detection in dual distribution systems, in integrity monitoring in a reverse osmosis process, and in microbial contamination detection in a groundwater based drinking water supply plant^15^,^21^,^28^. Loadings for emission and excitation spectra of all components identified in current research have been listed in Supporting Information (Table S3, S4 and S5). The distinctive components originated from algal excretion and EfOM identified in current research may provide a reference for warning a *M. aeruginosa* bloom or a contamination of water supply by wastewater in further application.

In terms of online monitoring, the remarkable correlation between fluorescence intensity measured and *F*_*max*_ of corresponding component around the peak location in the individual models (Table 4, and Table S1-2 in Supporting Information) implies that monitoring via a small number of simple fluorometers with appropriate wavelength selectivity (i.e. peak picking method) should capture essentially the same information as would online monitoring of full EEMs. Moreover, the sensitivity and accuracy of this peak monitoring strategy should be hardly affected by the peak shift, because of the relatively big high *R*^*2*^ area observed (Fig. 3 and Supplementary Fig. S7-8). But the relatively low *R*^*2*^ of some components in the mixture models (that were constructed for modeling NOM samples contaminated by EOM or EfOM) implies that during a contamination event (especially for those contaminant components that are spectrally overlapped with the original components), the peak monitoring strategy mentioned above may fail (as shown in Fig. 4). This overlap seems to be less possible when involving a single and unaltered DOM source. With all the considerations above, it is suggested that a regular check of unexpected fluorophore intrusion is necessary during the implementation of the peak monitoring strategy.

## Methods

### Sample description

In order to enable the diversity of an EEM dataset, a mixture of natural, industrial and manipulated samples were collected in this research. Samples were collected from an oligotrophic river dominated by terrestrial DOM derived from runoff, as well as effluent of a municipal wastewater treatment plant and organic matter secreted by algae which were regarded as the major sources of microbial derived DOM in aquatic environment.

Songhua River is located in the northeast part of China. 76 grab samples were collected during March to May, 2013. Samples were transported cold and filtered through 0.45 μm cellulose ester membrane (Taoyuan Co. Ltd., China), and then stored at 4 °C. These samples were referred to as natural organic matter (NOM).

Effluent organic matter (EfOM) was sampled from the Wenchang Wastewater Plant (Harbin, China), in which anaerobic-aerobic activated sludge treatment process was employed. The raw wastewater of WWTP was mainly municipal wastewater with a small portion of industrial wastewater. 62 EfOM samples were collected during one month. They were stored at 4 °C, and filtered through 0.45 μm cellulose ester membrane (Taoyuan Co. Ltd., China) prior to analysis.

Extracellular organic matter (EOM) excreted by algae was another microbial derived DOM in aquatic environment^17^. EOM was extracted from lab cultured *Microcystis aeruginosa* because of its prevalence in algae blooms in China^29^. *M. aeruginosa* was cultured in batch mode with BG11 medium at temperature of 25 °C with illumination of 5000 lx provided for 14h every day. Cultures were harvested at different phases during culture time of 20-40 days. Algal EOM was extracted by centrifuging the cell suspension at 10,000 rpm (11,179 *g*) and at 4 °C for 15 min and subsequently filtering the supernatant with 0.45 μm mix cellulose filter. In order to enable a larger EOM dataset for PARAFC analysis, ultrafiltration (UF) was conducted. Beforehand, the dissolved organic carbon (DOC) concentration of the extracted EOM was first measured with a TOC analyzer (multi N/C 2100S, Analytic Jena, Germany), and then the EOM solution was diluted to 5 ± 0.2 mg/L as DOC. Membrane filtration of the EOM was performed in a 400 mL unstirred dead-end cell (Amicon 8400, Millipore, USA). A flat sheet polyethersulfone (PES) UF membrane (OM 100076, Pall, USA) with molecular weight cut-off of 100 kDa was adopted. Nitrogen gas at a constant pressure of 0.03 MPa was used to drive the filtration. The feed, permeate, retentate, and backwash solution were collected during the filtration (*n* = 85). These samples contained identical components from the EOM but with different compositions, which enable a large enough dataset (20-100 samples) for PARAFAC analysis^19^.

### Fluorescence spectroscopy and PARAFAC modeling

The pH values of samples used for the EEM spectral analysis were all adjusted to 7.0 ± 0.1 beforehand. The ultraviolet-visible (UV-Vis) absorbances (200-800 nm in 1nm intervals) of all samples were measured using an UV-Vis spectrophotometer (Varian Cary 300 UV-Vis) beforehand. After that fluorescence of each sample was measured in a 1 cm cuvette using a Fluorescence Spectrophotometer (F7000, Hitachi, Japan) at room temperature (21 ± 1 °C). EEMs were generated by scanning over excitation wavelengths of 220-450 nm at an interval of 5 nm and emission wavelengths of 250-550 nm at an interval of 1 nm. Excitation and emission slit widths were both set at 5 nm. Photomultiplier tube (PMT) voltage at 700 V and scanning speed at 2400 nm/min were adopted. EEM of Milli-Q water sample was collected everyday throughout the experiment period. The average Raman scatter peak (λ_Ex/Em_ = 350/398 nm) value of 55.3 ± 3.2 arbitrary unit (A.U). (n = 119) showed the stability of the instrument^12^,^30^.

PARAFAC analysis uses an alternating least-squares algorithm to decompose the data signal into a set of trilinear terms and a residual array^9^:


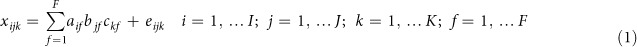


where *x*_*ijk*_ is the intensity of the *i*th sample at the *j*th variable (emission mode) and at the *k*th variable (excitation mode); *a*_*if*_ is directly proportional to the concentration of the *f*th analyte at emission wavelength *j*; *b*_*jf*_ is a scaled estimate of the emission spectrum of the *f*th analyte; *c*_*kf*_ is linearly proportional to the specific absorption coefficient (e.g., molar absorptivity) at excitation wavelength *k* and *e*_*ijk*_ is the residual noise, representing the variability not accounted for by the model.

The 3 individual EEM datasets (i.e. NOM, EOM, and EfOM), as well as a global dataset which encompassed all samples from different sources (223 EEMs) and two mixed datesets (62 NOM + 10 EOM and 62 NOM + 10 EfOM) were subjected to PARAFAC analysis respectively. PARAFAC modeling procedures were conducted according to the tutorial published by Murphy *et al*.^24^. The datasets were modeled using drEEM (http://www.models.life.ku.dk/drEEM) toolbox in Matlab® according to the tutorial, the appendix of drEEM toolbox, and the help files in drEEM.

Briefly, all EEMs were subjected to inner filter effect correction according to the UV-Vis absorbance data obtained before^24^,^31^. The EEMs were also Raman calibrated by normalizing to the area under the Raman scatter peak (Ex = 350 nm, Em = 381-426 nm) of Milli-Q water samples, run the same day. Then non-trilinear data were eliminated according to the tutorial. Since the global dataset and the mixed datasets encompassed large concentration gradients, each EEM in the datasets was normalized to its total signal before PARAFAC modeling. It allowed the model to focus on the chemical variations between samples rather than the magnitude of total signals, and it also increased the chance that minor peaks would be revealed. After the preprocessing above, PARAFAC modeling was conducted for each dataset. A series of PARAFAC models consisting of 3-7 components were generated. The number of fluorescence components was identified by a validation method including split half and residual analysis. After validating the model, the normalization was reversed by multiplying the scores by the sum of the squared value of all variables of the sample.

After PARAFAC modeling, vectors *a*, *b*, and *c* for each dataset were obtained for following analyses.

### Data analysis

In order to verify the representativeness and sensitivity of the global model, the excitation and emission spectrums of similar components from individual models and global model were compared. Tucker congruence coefficients were used to determine the similarity of two pairs of excitation and emission spectrum according to eq. (2):


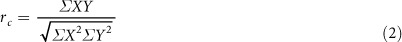


where *X* and *Y* were two Ex loadings (or Em loadings) from two PARAFAC models compared, and *r*_*c*_ was the congruence coefficient of the excitation spectrum (or the emission spectrum). A *r*_*c*_ value in the range of 0.85-0.94 has been seen as corresponding to a fair similarity, and values higher than 0.95 indicating that the factors can be considered to be equal^32^,^33^.

Previous studies proposed that monitoring the fluorescence data at the peak positions to estimate the PARAFAC *F*_max_^13^,^16^. However, the fluorescence overlap at the peak positions may deteriorate the accuracy of this estimation. To assess the accuracy and sensitivity of this online monitoring strategy, the PARAFAC intensity for each component (defined in eq. (3)) was regressed against the fluorescence intensities in the original EEMs. Linear regression was performed to obtain slope (*m*) and correlation coefficients (*R*^2^) as a function of wavelength.


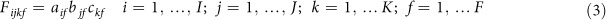


where *F*_*ijkf*_ is the calculated maximum fluorescence intensity of the *f*th component in the *i*th sample at the *j*th variable (emission mode) and at the *k*th variable (excitation mode). The *a*_*if*_, *b*_*jf*_, and *c*_*kf*_ were obtained from the PARAFAC model and defined in eq. (1). Thus, in the regression, for each wavelength pair in the EEM, an *i x 1* vector of *x*_*ijk*_ (defined in eq. (2)) was correlated with the *i x 1* vector of F_*ijkf*_, with this procedure repeated for each component in the PARAFAC model. The regression was conducted for each dataset (i.e. NOM, EOM, EfOM, the mixed datasets, and global dataset).

## Author Contributions

H.Y., H.L., & F.Q. designed the study. H.L., F.Q., & G.L. contributed to the critical revision of the article. H.Y., S.S., H.C, & Z.H. conducted the experiments, analyzed the data, prepared figures and drafted the article.

## Additional Information

**How to cite this article**: Yu, H. *et al*. Impact of dataset diversity on accuracy and sensitivity of parallel factor analysis model of dissolved organic matter fluorescence excitation-emission matrix. *Sci. Rep*. **5**, 10207; doi: 10.1038/srep10207 (2015).

## Supplementary Material

Supplementary Information

## Figures and Tables

**Table 1:** Description and wavelength positions of PARAFAC components in the Global model, and their comparisons with previously identified components.

This study		Previous studies		
Component	λ_ex_/λ_em_	λ_ex_/λ_em_	Description and source assignment	Reference
G1	230,305/414	<250,320/400	G2, Microbial humic-like fluorescence (wastewater)	13
		224,314/398	Component 1, humic-like substances (surface water)	34
G2	280/332	290/352	G6, protein, Tryptophan-like (wastewater)	25
		275/340	Peak A, tryptophan (*M. aeruginosa*)	13
		225,280/340	C3, protein like (surface water)	22
G3	245,285,335/420	250,340/438	Component 4, humic-like substances (*M. aeruginosa*)	27
		260,360/440	Component 3, humic-like substances (*M. aeruginosa*)	26
G4	225/332	<250/348	G5, protein, Tryptophan-like (wastewater)	13
		<224/344	Component 3, protein like (surface water)	35
G5	245,290/364	<250,290/360	C4, amino acids, free or protein bound (surface water)	16
		250,290/360	Component 4, protein-like substances (*M. aeruginosa*)	13
G6	265,365/472	<250,370/464	G1, Terrestrial humic-like fluorescence in high nutrient and wastewater impacted environments (wastewater)	9
		270,360/478	Component 3, humic-like (coastal water)	25
		270,360/470	C2, humic-like (surface water)	14
G7	265/314	270/300	G7, Protein, Tyrosine-like (wastewater)	26
		270/305	Component 3, tyrosine (lake)	13
		<300,280-380	Component 6, protein-like, microbial delivered (drinking water)	22

**Table 2:** Tucker correlation coefficients (*r*_*c*_) of similar components from global and individual models.

Global	NOM (Ex/Em)	EOM (Ex/Em)	EfOM (Ex/Em)
G1	**0.9052**/0.9826		0.9782/**0.9076**
G2	**0.9390**/**0.7315**	0.9994/0.9987	0.9908/0.9827
G3		0.9827/0.9638	
G4	0.9672/0.7670	0.9976/**0.8776**	0.9650/0.9790
G5		0.9637/0.9512	
G6	0.9779/**0.9337**	**0.9025**/**0.9078**	0.9563/0.9215
G7			0.9945/0.9885

*r*_*c*_ that is lower than 0.95 is featured in a **bold** type.

**Table 3:** Correlation coefficient (*R*^*2*^) and regression coefficient (*m*) of the linear regression of *F*_*max*_ in individual models versus the global model.

Sample		Components in global model						
		G1	G2	G3	G4	G5	G6	G7
NOM	*R*^*2*^	0.9610	**0.8070**		0.9795		0.9957	
	*m*	1.0353	0.5674		0.5192		0.4498	
EOM	*R*^*2*^		**0.6217**	0.9708	0.9271	0.9538	0.9795	
	*m*		0.8675	1.0334	0.3053	1.1283	0.8324	
EfOM	*R*^*2*^	**0.7117**	0.9383		0.9909		0.9883	0.9966
	*m*	0.8610	0.9476		0.9623		0.9056	0.9857

*R*^*2*^ that are significantly different from 1.0 are featured in a **bold** type.

**Table 4:** Correlation coefficient (*R*^*2*^) and regression coefficient (*m*) obtained from linear regression (*F*_*max*_ against original fluorescence intensity) with NOM dataset, NOM+EOM, and NOM+EfOM dataset.

Components		NOM1	NOM2	NOM3
Peak location(*λ*_*ex*_/*λ*_*em*_)		240,310/394	260,365/446	225,275/336
NOM dataset	*R*^*2*^	0.9794, 0.9785	0.9684,0.9543	0.9303, 0.9059
	*m*	0.9071, 0.8594	0.8428,0.8975	1.2316, 0.9320
NOM+EOM	*R*^*2*^	0.9812,0.9768	0.9578,0.9787	**0.7947, 0.8311**
	*m*	0.8606,0.5879	0.4950, 0.7984	0.7586, 0.9927
NOM + EfOM	*R*^*2*^	**0.8011, 0.8597**	**0.8137, 0.7790**	0.9354, **0.7564**
	*m*	0.1235, 0.1127	0.0677, 0.0803	0.7962, 0.8455

*R*^*2*^ that are significantly different from 1.0 are featured in a **bold** type.
