# Supplementary material for: Impact of dataset diversity on accuracy and sensitivity of parallel factor analysis model of dissolved organic matter fluorescence excitation-emission matrix
Source: Sci Rep. 2015 May 11;5:10207. doi: 10.1038/srep10207 (PMC4426691; doi:10.1038/srep10207)
Supplement: Supplementary Information [file srep10207-s1.doc]

# Supplementary information

# Of

# Impact of dataset diversity on accuracy and sensitivity of parallel factor analysis model of dissolved organic matter fluorescence excitation-emission matrix

Huarong Yu, Heng Liang^*^, Fangshu Qu, Zheng-shuang Han, Senlin Shao, Haiqing Chang, Guibai Li

*State Key Laboratory of Urban Water Resource and Environment (SKLUWRE), Harbin Institute of Technology, 73 Huanghe Road, Nangang District, Harbin, 150090, P.R. China*

* Corresponding author. Tel.: +86 451 86283001; Fax: +86 451 86283001.

E-mail address: huarongyu@gmail.com (Huarong Yu), hitliangheng@163.com (Heng Liang), qufangshu@163.com (Fangshu Qu), hustwater04_hzs@163.com (Zhuang-shuang Han), shaosenlin@gmail.com (Senlin Shao), changingchq@126.com (Haiqing Chang), hitsteven@gmail.com (Guibai Li).

## 1 Half split validation of global and individual models

**Figure S1** Split half validation of 3 components model (excitation to the left of emission spectra) with NOM dataset

**Figure S2** Split half validation of 5 components model (excitation to the left of emission spectra) with EOM dataset

**Figure S3** Split half validation of 5 components model (excitation to the left of emission spectra) with EfOM dataset.

**Figure S4** Split half validation of 7 components model (excitation to the left of emission spectra) with global dataset

**Figure S5** Split half validation of 6 components model (excitation to the left of emission spectra) with mixed dataset (76 NOM and 10 EOM samples).

**Figure S6** Split half validation of 6 components model (excitation to the left of emission spectra) with mixed dataset (76 NOM and 10 EfOM samples).

## 2 Correlation between *F_max_* and original EEM fluorescence intensity for each component in individual models

**Figure S7** Contour plot of each component, and correlation coefficient (*R^2^*) and regression coefficient (*m*) obtained via linear regression (*F_max_* against original fluorescence intensity) for each component in the NOM model.

**Table S1** Peak locations of components in NOM model, correlation coefficient (*R^2^*) and regression coefficient (*m*) at the peak locations.

| Component | Peak location (*λ_ex_*/*λ_em_*) | *R^2^* | *m* |
| --- | --- | --- | --- |
| NOM1 | 240,310/394 | 0.9794, 0.9785 | 0.9071, 0.8594 |
| NOM2 | 260/446 | 0.9684 | 0.8428 |
| NOM3 | 225,275/336 | 0.9303, **0.6959** | 1.2316, 0.9320 |

*R^2^* that are significantly different from 1.0 are featured in a **bold** type.

**Figure S8** Contour plot of each component, and correlation coefficient (*R^2^*) and regression coefficient (*m*) obtained via linear regression (*F_max_* against original fluorescence intensity) for each component in the NOM dipped by EOM model.

**Table S2** Peak locations of components in NOM dipped by EOM model, correlation coefficient (*R^2^*) and regression coefficient (*m*) at the peak locations.

| Component | Peak location (*λ_ex_*/*λ_em_*) | *R^2^* | *m* |
| --- | --- | --- | --- |
| NOM1 | 240 /394 | 0.9812 | 0.8606 |
| NOM2 | 260,365/446 | 0.9578, 0.9787 | 0.4950, 0.7984 |
| NOM3 | 275/336 | **0.7947** | 0.7586 |
| NOM3 | 225, /336 | **0.8311** | 0.9927 |

*R^2^* that are significantly different from 1.0 are featured in a **bold** type.

**Figure S9** Contour plot of each component, and correlation coefficient (*R^2^*) and regression coefficient (*m*) obtained via linear regression (*F_max_* against original fluorescence intensity) for each component in the NOM dipped by EfOM model.

**Table S3** Peak locations of components in NOM dipped by EOM model, correlation coefficient (*R^2^*) and regression coefficient (*m*) at the peak locations.

| Component | Peak location (*λ_ex_*/*λ_em_*) | *R^2^* | *m* |
| --- | --- | --- | --- |
| NOM1 | 245,315 /394 | **0.8011, 0.8597** | 0.1235, 0.1127 |
| NOM2 | 260,365/446 | **0.8137, 0.7790** | 0.0677, 0.0803 |
| NOM3 | 275/336 | 0.9354 | 0.7962 |
| NOM3 | 225, /336 | **0.7564** | 0.8455 |

*R^2^* that are significantly different from 1.0 are featured in a **bold** type.

**Figure S10** Contour plot of each component, and correlation coefficient (*R^2^*) and regression coefficient (*m*) obtained via linear regression (*F_max_* against original fluorescence intensity) for each component in the EOM model.

**Table S4** Peak locations of components in EOM model, correlation coefficient (*R^2^*) and regression coefficient (*m*) at the peak locations.

| Component | Peak location (*λ_ex_*/*λ_em_*) | *R^2^* | *m* |
| --- | --- | --- | --- |
| EOM1 | 240,285,330/414 | 0.9813, 0.9775, 0.9834 | 1.0051, 0.7921, 0.7887 |
| EOM2 | 220/330 | 0.9141 | 1.5541 |
| EOM3 | 280/330 | 0.9015 | 0.8669 |
| EOM4 | 255,360/434 | 0.9599, 0.9904 | 0.5096, 0.7325 |
| EOM5 | 250/364 | 0.9036 | 1.0638 |

**Figure S11** Contour plot of each component, and correlation coefficient (*R^2^*) and regression coefficient (*m*) obtained via linear regression (*F_max_* against original fluorescence intensity) for each component in the EfOM model.

**Table S5** Peak locations of components in EfOM model, correlation coefficient (*R^2^*) and regression coefficient (*m*) at the peak locations.

| Component | Peak location (*λ_ex_*/*λ_em_*) | *R^2^* | *m* |
| --- | --- | --- | --- |
| EfOM1 | 245,300/402 | 0.9606, 0.9870 | 0.9051, 0.9717 |
| EfOM2 | 225/338 | 0.9668 | 0.8384 |
| EfOM3 | 280/334 | 0.9120 | 0.5915 |
| EfOM4 | 270/316 | 0.9326 | 0.7697 |
| EfOM5 | 260,360/444 | 0.9907, 0.9816 | 0.4998, 0.7628 |

**Figure S12** Contour plot of each component in global model, and correlation coefficient (*R^2^*) and regression coefficient (*m*) obtained via linear regression (*F_max_* against original fluorescence intensity) for each component in global model.

**Table S5** Peak locations of components in the global model, correlation coefficient (*R^2^*) and regression coefficient (*m*) at the peak locations.

| Component | Peak location  (λ_ex_/λ_em_) | *R^2^* | *m* |
| --- | --- | --- | --- |
| G1 | **230/414** | **0.6340** | 0.7988 |
| G2 | 280/332 | 0.9455 | 0.8674 |
| G3 | **245,285**,335/420 | **0.6381**, **0.8553**, 0.9220 | 0.5949, 0.7553, 0.9143 |
| G4 | 225/332 | 0.9935 | 0.8799 |
| G5 | **245,290**/364 | **0.8286, 0.6428** | 0.7404, 0.4174 |
| G6 | 265,365/472 | 0.9208, 0.9251 | 0.5760, 0.5376 |
| G7 | **265/314** | **0.7997** | 0.6832 |
| Peak locations with *R^2^* < 0.9 are featured in a **bold** type. | | | |

*R^2^* that are significantly different from 1.0 are featured in a **bold** type.

## 3 Loading for global and individual PARAFAC model in this study

**Table S4** Loadings for emission (*λ_em_*) and excitation (*λ_ex_*) spectra of components in the global model

| *λ_em_* | G1 | G2 | G3 | G4 | G5 | G6 | G7 |
| --- | --- | --- | --- | --- | --- | --- | --- |
| 280 | 0.026661 | 0.005193 | 0 | 0 | 0 | 0 | 0.006509 |
| 284 | 0.013896 | 0.017631 | 0 | 0 | 0 | 0 | 0.013145 |
| 288 | 0.036267 | 0.039208 | 0.000729 | 0 | 0 | 0.000146 | 0.012339 |
| 292 | 0.058229 | 0.060957 | 0.00069 | 0 | 0 | 0.005245 | 0.013365 |
| 296 | 0.082507 | 0.082848 | 0 | 0.029046 | 0 | 0.013109 | 0.01628 |
| 300 | 0.106664 | 0.109978 | 0 | 0.218059 | 0 | 0.01599 | 0.020783 |
| 304 | 0.133077 | 0.141447 | 0 | 0.378237 | 0 | 0.016777 | 0.019485 |
| 308 | 0.168071 | 0.16744 | 0 | 0.381176 | 0.002357 | 0.01658 | 0.014878 |
| 312 | 0.203201 | 0.197298 | 0 | 0.403468 | 0.004217 | 0.017036 | 0.009641 |
| 316 | 0.238701 | 0.229628 | 0 | 0.414034 | 0.004785 | 0.023014 | 0.006776 |
| 320 | 0.269175 | 0.267334 | 0 | 0.360312 | 0.004938 | 0.038823 | 0.009005 |
| 324 | 0.294619 | 0.294091 | 0 | 0.290512 | 0.002091 | 0.05334 | 0.015951 |
| 328 | 0.307632 | 0.301067 | 0 | 0.230991 | 0 | 0.075473 | 0.026931 |
| 332 | 0.310976 | 0.301149 | 0.001101 | 0.174695 | 0 | 0.097941 | 0.037888 |
| 336 | 0.305469 | 0.300373 | 0.000936 | 0.128038 | 0 | 0.127114 | 0.046985 |
| 340 | 0.292247 | 0.289597 | 0 | 0.086429 | 0 | 0.169336 | 0.054169 |
| 344 | 0.272064 | 0.269626 | 0 | 0.061822 | 0 | 0.214849 | 0.056606 |
| 348 | 0.246452 | 0.244071 | 0 | 0.047674 | 0 | 0.256038 | 0.063945 |
| 352 | 0.219556 | 0.218637 | 0 | 0.026491 | 0 | 0.2777 | 0.076169 |
| 356 | 0.191564 | 0.193424 | 0 | 0.01123 | 0 | 0.2988 | 0.090473 |
| 360 | 0.164422 | 0.167489 | 0.014311 | 0.003744 | 0 | 0.302551 | 0.10683 |
| 364 | 0.138334 | 0.144668 | 0.021412 | 0.004781 | 0.00081 | 0.316175 | 0.119103 |
| 368 | 0.115346 | 0.122975 | 0.038518 | 0.000301 | 0.011545 | 0.299905 | 0.133763 |
| 372 | 0.094188 | 0.104039 | 0.058734 | 0 | 0.02108 | 0.277384 | 0.149983 |
| 376 | 0.075417 | 0.08742 | 0.074638 | 0 | 0.032235 | 0.257116 | 0.164916 |
| 380 | 0.059045 | 0.073475 | 0.091853 | 0 | 0.043908 | 0.238485 | 0.178479 |
| 384 | 0.046841 | 0.061306 | 0.113134 | 0 | 0.046406 | 0.221558 | 0.193158 |
| 388 | 0.036015 | 0.051501 | 0.138013 | 0 | 0.053658 | 0.195985 | 0.20612 |
| 392 | 0.027551 | 0.043228 | 0.164769 | 0 | 0.061442 | 0.169674 | 0.216263 |
| 396 | 0.020674 | 0.036583 | 0.191668 | 0 | 0.068082 | 0.144374 | 0.226464 |
| 400 | 0.015177 | 0.031776 | 0.214888 | 0 | 0.077972 | 0.121493 | 0.232153 |
| 404 | 0.011074 | 0.027367 | 0.238379 | 0 | 0.084653 | 0.099542 | 0.236273 |
| 408 | 0.008241 | 0.023556 | 0.257139 | 9.98E-05 | 0.092703 | 0.077965 | 0.239522 |
| 412 | 0.005992 | 0.020877 | 0.270851 | 0 | 0.102833 | 0.057789 | 0.239125 |
| 416 | 0.004429 | 0.018552 | 0.278324 | 0 | 0.113575 | 0.04048 | 0.237277 |
| 420 | 0.002866 | 0.017052 | 0.279971 | 0.001135 | 0.12554 | 0.026267 | 0.232742 |
| 424 | 0.001788 | 0.01623 | 0.277106 | 0.002757 | 0.136052 | 0.015208 | 0.227615 |
| 428 | 0.000605 | 0.017672 | 0.268819 | 0.00377 | 0.149462 | 0.007917 | 0.218117 |
| 432 | 0.000535 | 0.014825 | 0.257948 | 0.003856 | 0.160453 | 0.001979 | 0.210708 |
| 436 | 9.89E-05 | 0.015496 | 0.243241 | 0.003968 | 0.170575 | 0 | 0.201594 |
| 440 | 0 | 0.033037 | 0.223344 | 0.002894 | 0.184991 | 0 | 0.183746 |
| 444 | 0 | 0.021014 | 0.206349 | 0.003419 | 0.1883 | 0 | 0.177043 |
| 448 | 0 | 0.036653 | 0.186533 | 0.00261 | 0.194332 | 0 | 0.16455 |
| 452 | 0 | 0 | 0.159351 | 0.003342 | 0.215272 | 0.00327 | 0.128439 |
| 456 | 0 | 0 | 0.14223 | 0.002878 | 0.213004 | 0.004975 | 0.121238 |
| 460 | 0.001766 | 0.0022 | 0.125995 | 0.001743 | 0.203909 | 0.006905 | 0.121489 |
| 464 | 0 | 0 | 0.093136 | 0.001861 | 0.240985 | 0.006784 | 0.061062 |
| 468 | 0 | 0 | 0.079681 | 0.001426 | 0.230313 | 0.009996 | 0.059081 |
| 472 | 0 | 0 | 0.0561 | 0.001648 | 0.248731 | 0 | 0.023823 |
| 476 | 0 | 0 | 0.045993 | 0.001196 | 0.236112 | 0.001563 | 0.021646 |
| 480 | 0 | 0 | 0.035395 | 0.001689 | 0.2235 | 0.011478 | 0.023397 |
| 484 | 0 | 0 | 0.024905 | 0.000134 | 0.221813 | 0 | 0.00718 |
| 488 | 0 | 0 | 0.019435 | 0.00078 | 0.21191 | 0 | 0.007372 |
| 492 | 0 | 0 | 0.008773 | 0 | 0.202111 | 0 | 0 |
| 496 | 0 | 0 | 0.005326 | 0 | 0.190358 | 0 | 0 |
| 500 | 0.000695 | 0 | 0 | 0.001228 | 0.186259 | 0 | 0 |
| 504 | 0.00034 | 0 | 0.000538 | 0 | 0.155582 | 0 | 0 |
| 508 | 0.001716 | 0 | 0 | 0 | 0.144393 | 0 | 0 |
| 512 | 0.001541 | 0 | 0.000844 | 0 | 0.118498 | 0 | 0 |
| 516 | 0.001874 | 0 | 0 | 0 | 0.109263 | 0 | 0 |
| 520 | 0.007453 | 0 | 0 | 0 | 0.102566 | 0 | 0 |
| 524 | 0.001889 | 0 | 0.003575 | 0 | 0.076318 | 0 | 0 |
| 528 | 0.005071 | 0 | 0.000931 | 0 | 0.072044 | 0 | 0 |
| 532 | 0.001018 | 0 | 0.006828 | 0 | 0.050771 | 0 | 0 |
| 536 | 0.001937 | 0 | 0.005429 | 0 | 0.046491 | 0 | 0 |
| 540 | 0.014033 | 0 | 0.000539 | 0 | 0.045085 | 0 | 0 |
| 544 | 0 | 0 | 0.007389 | 0 | 0.029084 | 0 | 0 |
| 548 | 0.003871 | 0 | 0.005811 | 0 | 0.026066 | 0 | 0 |

| *λ_ex_* | G1 | G2 | G3 | G4 | G5 | G6 | G7 |
| --- | --- | --- | --- | --- | --- | --- | --- |
| 220 | 0 | 0.619282 | 0 | 0.133973 | 0 | 0 | 0.24643 |
| 225 | 0.082162 | 0.66736 | 0.020186 | 0.060561 | 0 | 0.02554 | 0.324467 |
| 230 | 0.172769 | 0.386286 | 0.083152 | 0.055039 | 0 | 0.108528 | 0.366383 |
| 235 | 0.14944 | 0.129506 | 0.15975 | 0.090163 | 0.04023 | 0.20556 | 0.345448 |
| 240 | 0.087337 | 0.050189 | 0.212988 | 0.152773 | 0.110544 | 0.267374 | 0.288219 |
| 245 | 0.064608 | 0.033058 | 0.20742 | 0.231623 | 0.215658 | 0.291034 | 0.234107 |
| 250 | 0.08487 | 0.028368 | 0.197889 | 0.295414 | 0.248968 | 0.284022 | 0.209432 |
| 255 | 0.138113 | 0.013733 | 0.174879 | 0.293678 | 0.282101 | 0.258509 | 0.193041 |
| 260 | 0.210269 | 0.001927 | 0.170213 | 0.339977 | 0.282419 | 0.231581 | 0.190742 |
| 265 | 0.294754 | 0 | 0.168821 | 0.361239 | 0.28924 | 0.221268 | 0.184374 |
| 270 | 0.380892 | 6.6E-06 | 0.180121 | 0.356896 | 0.279886 | 0.225841 | 0.176425 |
| 275 | 0.438992 | 0.006046 | 0.193771 | 0.334676 | 0.261779 | 0.231576 | 0.164725 |
| 280 | 0.445717 | 0.013028 | 0.20583 | 0.28231 | 0.231218 | 0.241467 | 0.151881 |
| 285 | 0.380208 | 0.012808 | 0.20918 | 0.246936 | 0.200096 | 0.250447 | 0.143635 |
| 290 | 0.267021 | 0.011809 | 0.197814 | 0.195832 | 0.172546 | 0.263658 | 0.13922 |
| 295 | 0.129631 | 0 | 0.172044 | 0.187361 | 0.150002 | 0.246482 | 0.145941 |
| 300 | 0.049816 | 0 | 0.151287 | 0.125336 | 0.135295 | 0.219105 | 0.152932 |
| 305 | 0.015832 | 0 | 0.149177 | 0.032552 | 0.126622 | 0.197335 | 0.15415 |
| 310 | 0.006541 | 0 | 0.160383 | 0.018966 | 0.121148 | 0.171927 | 0.150636 |
| 315 | 0 | 0 | 0.175746 | 0 | 0.11854 | 0.153414 | 0.142784 |
| 320 | 0 | 0 | 0.193685 | 0 | 0.118 | 0.130761 | 0.131257 |
| 325 | 0 | 0 | 0.209355 | 0 | 0.119618 | 0.107976 | 0.117857 |
| 330 | 0 | 0.00248 | 0.221828 | 0 | 0.12327 | 0.088878 | 0.101814 |
| 335 | 0 | 0 | 0.228844 | 0 | 0.127503 | 0.065994 | 0.084795 |
| 340 | 0 | 0 | 0.227832 | 0 | 0.131868 | 0.041261 | 0.068732 |
| 345 | 0 | 2.05E-05 | 0.220691 | 0 | 0.135974 | 0.018753 | 0.052568 |
| 350 | 0 | 0.00257 | 0.208331 | 0 | 0.139677 | 0.000819 | 0.03861 |
| 355 | 0 | 0 | 0.187751 | 0 | 0.146348 | 0 | 0.021097 |
| 360 | 0 | 0 | 0.163793 | 0 | 0.151092 | 0 | 0.008748 |
| 365 | 0 | 0.000154 | 0.13465 | 0 | 0.15286 | 0 | 0.00037 |
| 370 | 0 | 0.000673 | 0.1013 | 0 | 0.148143 | 0 | 0 |
| 375 | 0 | 0 | 0.069109 | 0 | 0.138825 | 0 | 0 |
| 380 | 0 | 0 | 0.043516 | 0 | 0.127514 | 0 | 0 |
| 385 | 0 | 0 | 0.025506 | 0 | 0.114975 | 0 | 0 |
| 390 | 0 | 8.11E-05 | 0.014572 | 0 | 0.103046 | 0 | 0 |
| 395 | 0 | 0 | 0.004989 | 0 | 0.091746 | 0 | 0 |
| 400 | 0 | 0 | 0 | 0 | 0.080885 | 0 | 0 |

**Table S5** Loadings for emission (*λ_em_*) and excitation (*λ_ex_*) spectra of components in the NOM model

| *λ_em_* | NOM 1 | NOM 2 | NOM 3 | *λ_ex_* | NOM 1 | NOM 2 | NOM 3 |
| --- | --- | --- | --- | --- | --- | --- | --- |
| 280 | 0 | 0.001932 | 0.018097 | 220 | 0.133116 | 0 | 0.348149 |
| 284 | 0 | 0 | 0.024445 | 225 | 0.212369 | 0 | 0.379583 |
| 288 | 0 | 0 | 0.04461 | 230 | 0.273852 | 0 | 0.348236 |
| 292 | 0 | 0 | 0.06741 | 235 | 0.319094 | 0.019721 | 0.239943 |
| 296 | 0 | 0 | 0.089243 | 240 | 0.331645 | 0.053969 | 0.159297 |
| 300 | 0 | 0 | 0.112415 | 245 | 0.299378 | 0.159492 | 0.136711 |
| 304 | 0 | 0 | 0.126916 | 250 | 0.270515 | 0.212692 | 0.138338 |
| 308 | 0 | 0.000141 | 0.138712 | 255 | 0.232712 | 0.275062 | 0.15648 |
| 312 | 0 | 0.002969 | 0.146271 | 260 | 0.212036 | 0.295298 | 0.177087 |
| 316 | 0 | 0.007161 | 0.15572 | 265 | 0.183461 | 0.318939 | 0.211872 |
| 320 | 0 | 0.008201 | 0.174704 | 270 | 0.161901 | 0.317707 | 0.249418 |
| 324 | 0 | 0.001711 | 0.203761 | 275 | 0.142467 | 0.303316 | 0.278597 |
| 328 | 0 | 1.85E-05 | 0.224922 | 280 | 0.135038 | 0.268127 | 0.279674 |
| 332 | 0.005444 | 0 | 0.235287 | 285 | 0.137149 | 0.22947 | 0.253236 |
| 336 | 0.01202 | 0 | 0.242563 | 290 | 0.143336 | 0.193481 | 0.212425 |
| 340 | 0.02478 | 0 | 0.240533 | 295 | 0.150682 | 0.164083 | 0.163178 |
| 344 | 0.034305 | 0 | 0.241008 | 300 | 0.157333 | 0.144702 | 0.126315 |
| 348 | 0.04901 | 0.001428 | 0.232818 | 305 | 0.162165 | 0.133047 | 0.098732 |
| 352 | 0.061544 | 0.008099 | 0.221151 | 310 | 0.162627 | 0.12654 | 0.080507 |
| 356 | 0.075954 | 0.014885 | 0.210094 | 315 | 0.162934 | 0.121333 | 0.05643 |
| 360 | 0.090508 | 0.023505 | 0.198663 | 320 | 0.157801 | 0.119439 | 0.04003 |
| 364 | 0.10584 | 0.029984 | 0.187849 | 325 | 0.150069 | 0.119286 | 0.02682 |
| 368 | 0.120501 | 0.037657 | 0.178595 | 330 | 0.139846 | 0.120601 | 0.017774 |
| 372 | 0.136274 | 0.044907 | 0.168911 | 335 | 0.130622 | 0.121191 | 0 |
| 376 | 0.150817 | 0.051974 | 0.161479 | 340 | 0.114547 | 0.123862 | 0 |
| 380 | 0.165702 | 0.059096 | 0.15334 | 345 | 0.097721 | 0.125594 | 0 |
| 384 | 0.179312 | 0.064511 | 0.14808 | 350 | 0.082424 | 0.126501 | 0 |
| 388 | 0.193336 | 0.070158 | 0.141423 | 355 | 0.067183 | 0.127544 | 0 |
| 392 | 0.202921 | 0.078397 | 0.137413 | 360 | 0.053466 | 0.127894 | 0 |
| 396 | 0.213386 | 0.0841 | 0.133532 | 365 | 0.040808 | 0.125603 | 0 |
| 400 | 0.220252 | 0.092523 | 0.128524 | 370 | 0.029993 | 0.119078 | 0 |
| 404 | 0.226389 | 0.099028 | 0.123497 | 375 | 0.019063 | 0.109892 | 0 |
| 408 | 0.230866 | 0.104841 | 0.120327 | 380 | 0.010865 | 0.09892 | 0 |
| 412 | 0.232083 | 0.112077 | 0.116231 | 385 | 0.00474 | 0.087586 | 0 |
| 416 | 0.232415 | 0.117533 | 0.112619 | 390 | 0.001137 | 0.07724 | 0 |
| 420 | 0.230389 | 0.123779 | 0.108722 | 395 | 0 | 0.065304 | 0 |
| 424 | 0.226462 | 0.129866 | 0.104345 | 400 | 0 | 0.055066 | 0 |
| 428 | 0.223687 | 0.136539 | 0.093995 |  | | | |
| 432 | 0.219261 | 0.144762 | 0.080709 |  | | | |
| 436 | 0.212484 | 0.14932 | 0.077004 |  | | | |
| 440 | 0.20512 | 0.154385 | 0.068671 |  | | | |
| 444 | 0.195025 | 0.161624 | 0.057489 |  | | | |
| 448 | 0.186086 | 0.161241 | 0.055344 |  | | | |
| 452 | 0.167039 | 0.196877 | 0 |  | | | |
| 456 | 0.15917 | 0.191192 | 0 |  | | | |
| 460 | 0.156201 | 0.179372 | 0 |  | | | |
| 464 | 0.102694 | 0.222615 | 0 |  | | | |
| 468 | 0.098984 | 0.210193 | 0 |  | | | |
| 472 | 0.043914 | 0.252105 | 0 |  | | | |
| 476 | 0.040565 | 0.238297 | 0 |  | | | |
| 480 | 0.044396 | 0.221891 | 0 |  | | | |
| 484 | 0.012706 | 0.234193 | 0 |  | | | |
| 488 | 0.012563 | 0.222566 | 0 |  | | | |
| 492 | 0 | 0.216246 | 0 |  | | | |
| 496 | 0 | 0.203071 | 0 |  | | | |
| 500 | 0 | 0.195217 | 0 |  | | | |
| 504 | 0 | 0.169895 | 0 |  | | | |
| 508 | 0 | 0.159581 | 0 |  | | | |
| 512 | 0 | 0.136687 | 0 |  | | | |
| 516 | 0 | 0.126984 | 0 |  | | | |
| 520 | 0 | 0.124629 | 0 |  | | | |
| 524 | 0 | 0.096156 | 0.00034 |  | | | |
| 528 | 0 | 0.091394 | 0.000429 |  | | | |
| 532 | 0 | 0.069695 | 0.000272 |  | | | |
| 536 | 0 | 0.06422 | 0.000407 |  | | | |
| 540 | 0 | 0.065673 | 0.002228 |  | | | |
| 544 | 0 | 0.0433 | 0 |  | | | |
| 548 | 0 | 0.041058 | 0 |  | | | |

**Table S6** Loadings for emission (*λ_em_*) and excitation (*λ_ex_*) spectra of components in the EOM model

| *λ_em_* | EOM 1 | EOM 2 | EOM 3 | EOM 4 | EOM 5 |
| --- | --- | --- | --- | --- | --- |
| 280 | 0.030115 | 0.002913 | 0 | 0 | 0 |
| 284 | 0.014592 | 0.016183 | 0 | 0 | 0.000198 |
| 288 | 0.040494 | 0.045212 | 0 | 0 | 0.001406 |
| 292 | 0.06448 | 0.07204 | 0 | 0 | 0.005689 |
| 296 | 0.089456 | 0.095215 | 0 | 0 | 0.01552 |
| 300 | 0.119545 | 0.120088 | 0.002264 | 0 | 0.044217 |
| 304 | 0.148561 | 0.14866 | 0 | 0.007342 | 0.067419 |
| 308 | 0.18259 | 0.176534 | 0 | 0.006266 | 0.071003 |
| 312 | 0.216914 | 0.205552 | 0 | 0.005417 | 0.074679 |
| 316 | 0.250801 | 0.235303 | 0 | 0.004847 | 0.077422 |
| 320 | 0.279975 | 0.26584 | 0.007337 | 0 | 0.073382 |
| 324 | 0.302728 | 0.289036 | 0.00593 | 0 | 0.070824 |
| 328 | 0.312772 | 0.298457 | 0.013525 | 0 | 0.066967 |
| 332 | 0.312556 | 0.300439 | 0.022409 | 0 | 0.067373 |
| 336 | 0.303401 | 0.295899 | 0.028582 | 0 | 0.089633 |
| 340 | 0.285343 | 0.285649 | 0.03233 | 0 | 0.143976 |
| 344 | 0.260999 | 0.268498 | 0.022483 | 0 | 0.231395 |
| 348 | 0.232668 | 0.243142 | 0.023129 | 0 | 0.29992 |
| 352 | 0.204881 | 0.216909 | 0.036489 | 0 | 0.301294 |
| 356 | 0.176849 | 0.191679 | 0.05029 | 0 | 0.302643 |
| 360 | 0.149879 | 0.166646 | 0.064777 | 0 | 0.304056 |
| 364 | 0.12531 | 0.143474 | 0.075196 | 0 | 0.320364 |
| 368 | 0.104429 | 0.121646 | 0.095135 | 0 | 0.297 |
| 372 | 0.086569 | 0.102794 | 0.11912 | 0 | 0.263203 |
| 376 | 0.070589 | 0.085922 | 0.139029 | 0 | 0.2335 |
| 380 | 0.056492 | 0.071284 | 0.157502 | 0.004316 | 0.210842 |
| 384 | 0.045651 | 0.059363 | 0.17549 | 0.0106 | 0.192946 |
| 388 | 0.03615 | 0.04967 | 0.192013 | 0.031347 | 0.164387 |
| 392 | 0.028548 | 0.041559 | 0.209654 | 0.053343 | 0.13759 |
| 396 | 0.021977 | 0.034857 | 0.22433 | 0.079175 | 0.115102 |
| 400 | 0.016335 | 0.029798 | 0.235497 | 0.107028 | 0.097602 |
| 404 | 0.012132 | 0.025416 | 0.245886 | 0.134274 | 0.081999 |
| 408 | 0.009293 | 0.021473 | 0.252611 | 0.159868 | 0.067696 |
| 412 | 0.006774 | 0.018718 | 0.255504 | 0.183828 | 0.054859 |
| 416 | 0.004914 | 0.016377 | 0.253937 | 0.205069 | 0.045474 |
| 420 | 0.003262 | 0.014644 | 0.248535 | 0.222481 | 0.038975 |
| 424 | 0.002159 | 0.013007 | 0.240033 | 0.235301 | 0.035573 |
| 428 | 0.000812 | 0.014396 | 0.229412 | 0.245327 | 0.033492 |
| 432 | 0.000679 | 0.010893 | 0.216937 | 0.250692 | 0.033909 |
| 436 | 0.000195 | 0.011523 | 0.203563 | 0.252685 | 0.033998 |
| 440 | 0 | 0.030101 | 0.189856 | 0.25276 | 0.028752 |
| 444 | 0 | 0.010719 | 0.173878 | 0.245036 | 0.036391 |
| 448 | 0 | 0.026419 | 0.159325 | 0.238069 | 0.033237 |
| 452 | 0.000656 | 0.00046 | 0.142212 | 0.227956 | 0.039822 |
| 456 | 0.000707 | 0.000964 | 0.128445 | 0.216061 | 0.041312 |
| 460 | 0.001522 | 0.011323 | 0.111466 | 0.203941 | 0.048183 |
| 464 | 0.000785 | 0 | 0.101992 | 0.19296 | 0.039979 |
| 468 | 0.000811 | 0 | 0.089557 | 0.178438 | 0.045721 |
| 472 | 0.000854 | 0 | 0.077138 | 0.171066 | 0.035947 |
| 476 | 0.000724 | 0 | 0.067784 | 0.15732 | 0.037811 |
| 480 | 0 | 0 | 0.056348 | 0.142794 | 0.062174 |
| 484 | 0.001781 | 0 | 0.050182 | 0.138893 | 0.025359 |
| 488 | 0.001259 | 0 | 0.042908 | 0.12819 | 0.035095 |
| 492 | 0.004252 | 0 | 0.037476 | 0.120256 | 0.006816 |
| 496 | 0.004067 | 0 | 0.033023 | 0.109981 | 0.009169 |
| 500 | 0.004426 | 0 | 0.020475 | 0.106175 | 0.036186 |
| 504 | 0.005026 | 0 | 0.026339 | 0.085404 | 0 |
| 508 | 0.007023 | 0 | 0.022752 | 0.078744 | 0 |
| 512 | 0.004441 | 0 | 0.020521 | 0.063488 | 0 |
| 516 | 0.004931 | 0 | 0.018264 | 0.057444 | 0 |
| 520 | 0.013303 | 0 | 0.013078 | 0.055577 | 0 |
| 524 | 0.003487 | 0 | 0.01576 | 0.039662 | 0 |
| 528 | 0.008042 | 0 | 0.012811 | 0.036385 | 0 |
| 532 | 0.002083 | 0 | 0.013236 | 0.027364 | 0 |
| 536 | 0.003358 | 0 | 0.011576 | 0.024391 | 0 |
| 540 | 0.019794 | 0 | 0.005754 | 0.022503 | 0 |
| 544 | 0.000648 | 0 | 0.009875 | 0.016261 | 0 |
| 548 | 0.006295 | 0 | 0.007686 | 0.013759 | 0 |

| *λ_ex_* | EOM 1 | EOM 2 | EOM 3 | EOM 4 | EOM 5 |
| --- | --- | --- | --- | --- | --- |
| 220 | 0 | 0.585896 | 0.006797 | 0 | 0.017516 |
| 225 | 0.053726 | 0.668655 | 0.034578 | 0 | 0.048012 |
| 230 | 0.120913 | 0.408284 | 0.103955 | 0 | 0.126118 |
| 235 | 0.111696 | 0.15043 | 0.171393 | 0.043822 | 0.231671 |
| 240 | 0.075508 | 0.056959 | 0.216184 | 0.096591 | 0.315973 |
| 245 | 0.067644 | 0.028546 | 0.200532 | 0.174697 | 0.375175 |
| 250 | 0.095288 | 0.025068 | 0.186965 | 0.202449 | 0.378089 |
| 255 | 0.144932 | 0.026357 | 0.159818 | 0.23081 | 0.346124 |
| 260 | 0.217847 | 0.030723 | 0.163843 | 0.227024 | 0.272753 |
| 265 | 0.305308 | 0.038111 | 0.170481 | 0.230721 | 0.217088 |
| 270 | 0.388314 | 0.048717 | 0.188738 | 0.224283 | 0.201157 |
| 275 | 0.445112 | 0.056762 | 0.203763 | 0.221865 | 0.195908 |
| 280 | 0.449452 | 0.060547 | 0.218026 | 0.205474 | 0.204287 |
| 285 | 0.382002 | 0.048148 | 0.225917 | 0.181401 | 0.212244 |
| 290 | 0.266917 | 0.029416 | 0.220877 | 0.144529 | 0.231123 |
| 295 | 0.132328 | 0 | 0.204437 | 0.102066 | 0.185682 |
| 300 | 0.053333 | 0 | 0.195206 | 0.057729 | 0.134822 |
| 305 | 0.019172 | 0 | 0.195017 | 0.03937 | 0.111024 |
| 310 | 0.013126 | 0 | 0.203293 | 0.03891 | 0.085968 |
| 315 | 2.99E-05 | 0 | 0.214338 | 0.046288 | 0.074643 |
| 320 | 0 | 0 | 0.223217 | 0.064366 | 0.058013 |
| 325 | 0 | 0.002317 | 0.227345 | 0.089514 | 0.040149 |
| 330 | 7.85E-05 | 0.007775 | 0.230359 | 0.114492 | 0.027006 |
| 335 | 0 | 0.001587 | 0.221165 | 0.148202 | 0.01576 |
| 340 | 0 | 0.001862 | 0.202166 | 0.183801 | 0 |
| 345 | 0 | 0.001422 | 0.175347 | 0.217103 | 0 |
| 350 | 0 | 0.002237 | 0.149035 | 0.240034 | 0 |
| 355 | 0 | 0 | 0.119589 | 0.257333 | 0 |
| 360 | 0 | 0 | 0.091837 | 0.262782 | 0 |
| 365 | 0 | 8.65E-05 | 0.06358 | 0.254329 | 0 |
| 370 | 0 | 0.000537 | 0.037388 | 0.22938 | 0 |
| 375 | 0 | 1.57E-05 | 0.004875 | 0.209064 | 0 |
| 380 | 0 | 3.69E-05 | 0 | 0.163819 | 0 |
| 385 | 0 | 6.84E-05 | 0 | 0.125666 | 0 |
| 390 | 0 | 0.000147 | 0 | 0.099663 | 8.11E-05 |
| 395 | 0 | 0 | 0 | 0.078681 | 0 |
| 400 | 0 | 3.15E-07 | 0 | 0.062517 | 0 |

**Table S7** Loadings for emission (*λ_em_*) and excitation (*λ_ex_*) spectra of components in the EfOM model

| *λ_em_* | EfOM 1 | EfOM 2 | EfOM 3 | EfOM 4 | EfOM 5 |
| --- | --- | --- | --- | --- | --- |
| 280 | 0.001685 | 0.013006 | 0 | 0.021602 | 0 |
| 284 | 0 | 0.022196 | 0.004329 | 0.011352 | 0 |
| 288 | 0 | 0.033951 | 0.006738 | 0.023422 | 0 |
| 292 | 0.003627 | 0.048819 | 0.009152 | 0.03886 | 0 |
| 296 | 0.042502 | 0.06637 | 0.011135 | 0.0586 | 0 |
| 300 | 0.21158 | 0.086587 | 0.014711 | 0.084746 | 0 |
| 304 | 0.354658 | 0.113614 | 0.012272 | 0.113707 | 0.003096 |
| 308 | 0.362778 | 0.138337 | 0.002322 | 0.148142 | 0.007553 |
| 312 | 0.387056 | 0.168835 | 0 | 0.181472 | 0.005404 |
| 316 | 0.40062 | 0.205006 | 0 | 0.217294 | 0.001072 |
| 320 | 0.357312 | 0.253186 | 0 | 0.245887 | 0.002598 |
| 324 | 0.298621 | 0.288068 | 0 | 0.273575 | 0.002667 |
| 328 | 0.247989 | 0.297061 | 0.010716 | 0.293878 | 0 |
| 332 | 0.198681 | 0.299027 | 0.026803 | 0.303404 | 0 |
| 336 | 0.156951 | 0.30466 | 0.044102 | 0.301936 | 0 |
| 340 | 0.120755 | 0.293992 | 0.064384 | 0.294156 | 0 |
| 344 | 0.099625 | 0.268228 | 0.08288 | 0.280795 | 0 |
| 348 | 0.086106 | 0.241834 | 0.103471 | 0.260936 | 0 |
| 352 | 0.064323 | 0.219741 | 0.121386 | 0.240201 | 0 |
| 356 | 0.048124 | 0.196543 | 0.140541 | 0.216783 | 0 |
| 360 | 0.037732 | 0.171676 | 0.160128 | 0.192325 | 0 |
| 364 | 0.036215 | 0.149702 | 0.178939 | 0.166072 | 0 |
| 368 | 0.028131 | 0.130395 | 0.194972 | 0.143382 | 0 |
| 372 | 0.018845 | 0.114795 | 0.210396 | 0.121955 | 0.00093 |
| 376 | 0.014901 | 0.101381 | 0.218092 | 0.100416 | 0.015226 |
| 380 | 0.013642 | 0.090542 | 0.223393 | 0.079583 | 0.034722 |
| 384 | 0.011879 | 0.081044 | 0.23091 | 0.063539 | 0.044811 |
| 388 | 0.007265 | 0.07475 | 0.23313 | 0.049025 | 0.063603 |
| 392 | 0.003138 | 0.069384 | 0.233258 | 0.037237 | 0.083945 |
| 396 | 0.001843 | 0.06542 | 0.233393 | 0.025969 | 0.103002 |
| 400 | 0.002044 | 0.062281 | 0.231419 | 0.01652 | 0.122657 |
| 404 | 0.001694 | 0.059302 | 0.228033 | 0.008467 | 0.140816 |
| 408 | 0.000193 | 0.057102 | 0.22311 | 0.003298 | 0.156322 |
| 412 | 0 | 0.054931 | 0.215369 | 0 | 0.171422 |
| 416 | 0 | 0.052516 | 0.204509 | 0 | 0.18508 |
| 420 | 0 | 0.050799 | 0.192969 | 0 | 0.197761 |
| 424 | 0 | 0.050499 | 0.182145 | 0 | 0.207509 |
| 428 | 0 | 0.050169 | 0.17084 | 0 | 0.216751 |
| 432 | 0 | 0.048301 | 0.160215 | 0 | 0.223786 |
| 436 | 0.000624 | 0.047423 | 0.149777 | 0 | 0.228197 |
| 440 | 0.000689 | 0.054904 | 0.13652 | 0 | 0.232891 |
| 444 | 0.001648 | 0.054063 | 0.126971 | 0 | 0.231482 |
| 448 | 0.001724 | 0.05862 | 0.116387 | 0 | 0.228661 |
| 452 | 0.006441 | 0.004084 | 0.105038 | 0.008798 | 0.222849 |
| 456 | 0.006279 | 0.004327 | 0.096907 | 0.00974 | 0.215145 |
| 460 | 0.005422 | 0.010929 | 0.088863 | 0.010957 | 0.205422 |
| 464 | 0.006209 | 0 | 0.074343 | 0.010217 | 0.2036 |
| 468 | 0.006097 | 0 | 0.068521 | 0.010913 | 0.191404 |
| 472 | 0.005521 | 0 | 0.050894 | 0.01111 | 0.189043 |
| 476 | 0.005225 | 0 | 0.046055 | 0.01097 | 0.175905 |
| 480 | 0.006572 | 0 | 0.045072 | 0.01018 | 0.163679 |
| 484 | 0.004478 | 0 | 0.026447 | 0.011646 | 0.160459 |
| 488 | 0.004767 | 0 | 0.024784 | 0.011254 | 0.149461 |
| 492 | 0.003058 | 0 | 0.010404 | 0.014469 | 0.141807 |
| 496 | 0.002912 | 0 | 0.009307 | 0.013959 | 0.13117 |
| 500 | 0.004497 | 0 | 0.007907 | 0.014404 | 0.122837 |
| 504 | 0.001189 | 0 | 0.001158 | 0.015621 | 0.107755 |
| 508 | 0.001725 | 0 | 0.000275 | 0.015668 | 0.099211 |
| 512 | 0 | 0 | 0 | 0.014247 | 0.083311 |
| 516 | 0 | 0 | 0 | 0.013485 | 0.075584 |
| 520 | 0.001691 | 0 | 0 | 0.016212 | 0.068904 |
| 524 | 0 | 0 | 0 | 0.010103 | 0.056542 |
| 528 | 0 | 0 | 0 | 0.011607 | 0.050663 |
| 532 | 0 | 0 | 0.000233 | 0.005771 | 0.042258 |
| 536 | 0 | 0 | 0 | 0.006013 | 0.037901 |
| 540 | 8.63E-06 | 0 | 0 | 0.014141 | 0.031792 |
| 544 | 0 | 0.000218 | 0.002136 | 2.64E-05 | 0.027217 |
| 548 | 0 | 0.000273 | 0.00061 | 0.003449 | 0.024277 |

| *λ_em_* | EfOM 1 | EfOM 2 | EfOM 3 | EfOM 4 | EfOM 5 |
| --- | --- | --- | --- | --- | --- |
| 220 | 0.165753 | 0.552216 | 0.034141 | 0 | 0 |
| 225 | 0.109532 | 0.639985 | 0.083161 | 0.033861 | 0 |
| 230 | 0.072939 | 0.442793 | 0.165587 | 0.148535 | 0 |
| 235 | 0.086872 | 0.186897 | 0.236492 | 0.163669 | 0.067684 |
| 240 | 0.147855 | 0.062823 | 0.270989 | 0.119964 | 0.130017 |
| 245 | 0.223398 | 0.019467 | 0.256207 | 0.109344 | 0.194329 |
| 250 | 0.285499 | 0.007712 | 0.233167 | 0.12875 | 0.219532 |
| 255 | 0.287989 | 0 | 0.203831 | 0.175768 | 0.242637 |
| 260 | 0.335242 | 0 | 0.192735 | 0.238954 | 0.242866 |
| 265 | 0.359671 | 0.020894 | 0.190162 | 0.311465 | 0.24117 |
| 270 | 0.357795 | 0.060314 | 0.194199 | 0.373131 | 0.228613 |
| 275 | 0.337297 | 0.100496 | 0.196829 | 0.411186 | 0.210204 |
| 280 | 0.288096 | 0.12463 | 0.202618 | 0.4075 | 0.182575 |
| 285 | 0.249123 | 0.113681 | 0.20971 | 0.356833 | 0.154826 |
| 290 | 0.195257 | 0.082683 | 0.218506 | 0.272222 | 0.127835 |
| 295 | 0.175252 | 0.024959 | 0.219942 | 0.176558 | 0.110228 |
| 300 | 0.113681 | 0 | 0.218995 | 0.103577 | 0.101175 |
| 305 | 0.028397 | 0 | 0.218349 | 0.05653 | 0.099279 |
| 310 | 0.016695 | 0 | 0.213509 | 0.032626 | 0.106178 |
| 315 | 0.001103 | 0.000561 | 0.211917 | 0.011089 | 0.112973 |
| 320 | 0 | 0.002164 | 0.202361 | 0 | 0.127086 |
| 325 | 0 | 0 | 0.185925 | 0 | 0.145977 |
| 330 | 0 | 0.001267 | 0.166333 | 0 | 0.165242 |
| 335 | 0 | 0 | 0.14372 | 0 | 0.182425 |
| 340 | 0 | 0 | 0.119795 | 0 | 0.195359 |
| 345 | 0 | 0 | 0.094794 | 0 | 0.205061 |
| 350 | 0 | 0 | 0.073296 | 0 | 0.21006 |
| 355 | 0 | 0 | 0.051344 | 0 | 0.213831 |
| 360 | 0 | 0 | 0.03288 | 0 | 0.21392 |
| 365 | 0 | 0 | 0.016051 | 0 | 0.209521 |
| 370 | 0 | 0.000384 | 0.003267 | 0 | 0.196755 |
| 375 | 0 | 0 | 0 | 0 | 0.172638 |
| 380 | 0 | 0 | 0 | 0 | 0.147538 |
| 385 | 0 | 0 | 0 | 0.000129 | 0.12509 |
| 390 | 0 | 0 | 0 | 0.000351 | 0.107492 |
| 395 | 0 | 0 | 0 | 0.000139 | 0.092237 |
| 400 | 1.57E-07 | 0 | 0 | 0.00012 | 0.079992 |
